# Supplementary figures and images for: Analysis and Comparison of Vector Space and Metric Space Representations in QSAR Modeling
Source: Molecules. 2019 Apr 30;24(9):1698. doi: 10.3390/molecules24091698 (PMC6539555; doi:10.3390/molecules24091698)

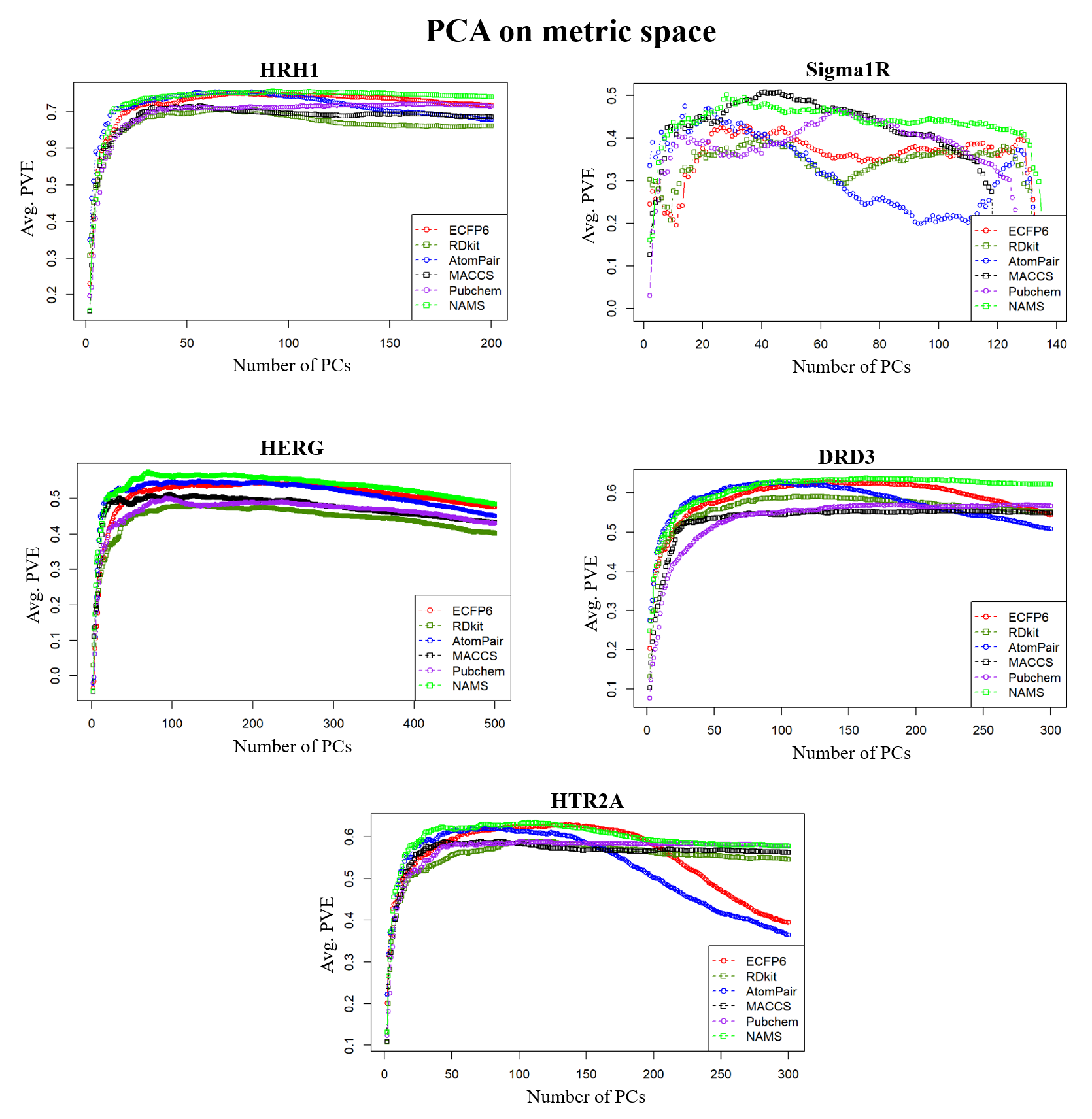

Supplement: Supplementary file 1 [file molecules-24-01698-s001.zip › Additional file 1/Figure S1.png]
